# Enhancing charge extraction in inverted perovskite solar cells contacts *via* ultrathin graphene:fullerene composite interlayers†

**DOI:** 10.1039/d2ta07512a

**Published:** 2022-11-30

**Authors:** Andrea Zanetta, Isabella Bulfaro, Fabiola Faini, Matteo Manzi, Giovanni Pica, Michele De Bastiani, Sebastiano Bellani, Marilena Isabella Zappia, Gabriele Bianca, Luca Gabatel, Jaya-Kumar Panda, Antonio Esaú Del Rio Castillo, Mirko Prato, Simone Lauciello, Francesco Bonaccorso, Giulia Grancini

**Affiliations:** a Department of Chemistry & INSTM, University of Pavia Via T. Taramelli 14 27100 Pavia Italy giulia.grancini@unipv.it; b BeDimensional S.p.A Via Lungotorrente Secca 30R 16163 Genova Italy; c Graphene Labs, Istituto Italiano di Tecnologia Via Morego 30 16163 Genova Italy; d Dipartimento di Chimica e Chimica Industriale, Università degli Studi di Genova Via Dodecaneso 31 16146 Genoa Italy; e Department of Mechanical Engineering – DIME, University of Genoa Via Opera Pia 15 16145 Genova Italy; f Materials Characterization Facility, Istituto Italiano di Tecnologia Via Morego 30 16163 Genova Italy; g Electron Microscopy Facility, Istituto Italiano di Tecnologia Via Morego 30 16163 Genova Italy

## Abstract

Improving the perovskite/electron-transporting layer (ETL) interface is a crucial task to boost the performance of perovskite solar cells (PSCs). This is utterly fundamental in an inverted (p–i–n) configuration using fullerene-based ETLs. Here, we propose a scalable strategy to improve fullerene-based ETLs by incorporating high-quality few-layer graphene flakes (GFs), industrially produced through wet-jet milling exfoliation of graphite, into phenyl-C61-butyric acid methyl ester (PCBM). Our new composite ETL (GF:PCBM) can be processed into an ultrathin (∼10 nm), pinhole-free film atop the perovskite. We find that the presence of GFs in the PCBM matrix reduces defect-mediated recombination, while creating preferential paths for the extraction of electrons towards the current collector. The use of our GF-based composite ETL resulted in a significant enhancement in the open circuit voltage and fill factor of triple cation-based inverted PSCs, boosting the power conversion efficiency from ∼19% up to 20.8% upon the incorporation of GFs into the ETL.

## Abstract 2

Improving the perovskite/electron-transporting layer (ETL) interface is a crucial task to boost the performance of perovskite solar cells (PSCs).

## Graphical abstract

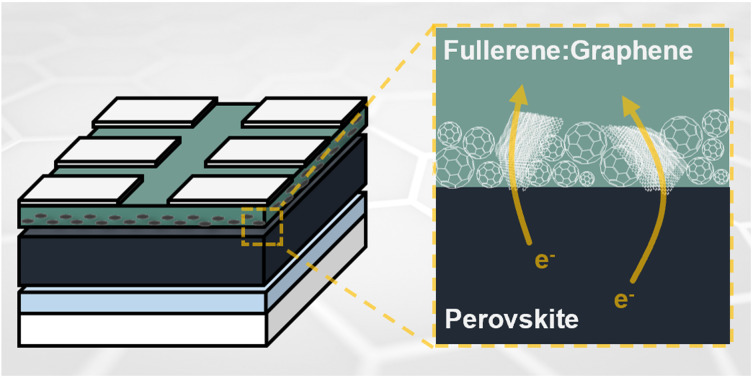

**10th anniversary statement**

Over the past ten years, the *Journal of Materials Chemistry A* has been the reference point for the progress of materials science. Particularly looking at photovoltaics, the perovskite solar cell community largely benefitted from the work published in *JMCA* since its beginning. Even today, *JMCA* is considered for the divulgation of high impact results. In this direction, we present here a novel contact interface for p–i–n perovskite solar cells, embedding graphene flakes into a fullerene matrix. Our results, supported by the reach of *JMCA*, will tailor new concepts of contact engineering towards more efficient and more stable perovskite solar cells.

## Introduction

Interfaces are fundamental elements in solar cells to achieve highly performing devices. The interface is the region of space where charge carriers, either holes or electrons, are extracted to reach external contacts.^1^ However, this is also the place where carriers trapping and recombination occurs, ultimately limiting the device operation.^2^ This is a particularly critical aspect for thin-film photovoltaic technologies, where “the interface is the device”,^3^ and minimizing contact losses is pivotal.^4–7^ Among thin-film photovoltaics, perovskite solar cells (PSCs) are one of the most promising technologies to disrupt the current Si-based photovoltaic market.^8–10^ PSCs can be constructed in the form of either standard or inverted configuration, also named n–i–p and p–i–n structures, respectively. Even though n–i–p configurations shine because of their remarkable power conversion efficiency (PCE) approaching 26%, they lack stability and employ materials that are not compatible with industrial production.^11–14^ Conversely, p–i–n configurations typically exhibit lower PCEs (up to ∼25.3%), but succeed in passing harsh accelerated stability tests and employ cost-effective materials.^15–18^ The main reason for the limited PCE of p–i–n configurations is found to be an inadequate material energy level alignment at the perovskite/electron-transporting layer (ETL) interface.^18,19^ Indeed, the most common p–i–n configurations rely on ETLs based on fullerenes (*e.g.*, C60 or phenyl-C61-butyric acid methyl ester PCBM) that are characterized by a poor energy alignment with the perovskite conduction band, leading to low charge extraction, which translates into high voltage losses.^20–22^ Unfortunately, alternative ETL materials to fullerenes are limited by the constraints imposed by the perovskite in terms of solvent, temperature, and processing compatibility.^23^ Not surprisingly, several studies have focused on the development of innovative fullerene derivatives^24^ and on the improvement of the interfaces between the perovskite and fullerenes, the processing of the latter being a technologically viable solution for large-area PSCs.^25^ In this context, the use of perovskites with low dimensionalities^15,16,26–28^ and the fabrication of heterostructures *via* the addition of inorganic interlayers^19,29,30^ represent strategies of utmost importance to build effective perovskite/ETL interfaces for efficient p–i–n devices. The use of graphene and related 2D materials at perovskite/charge-transporting layer interfaces is also an established approach to improve PSC performances, as shown by pioneering/major studies on n–i–p devices by incorporating graphene into oxide-based ETLs.^31–37^ Concerning the p–i–n configuration, reduced graphene oxide has been evaluated as an additive in PCBM-based ETLs.^38,39^ However, a lack of control on graphene quality in terms of chemical purity and structural and morphological properties impeded a rational understanding and optimization of such interface.^40^ For instance, graphene derivatives, such as reduced graphene oxide, exhibit a corrugated surface folded on itself, making its integration into the polymeric layer with a nanometric thickness challenging.^41,42^ Also, reduced graphene oxide and other graphene derivatives have been applied both on the HTL and ETL sides, causing confusion on their actual functionalities.^40^ To provide reliable insights into their functional role in PSC components, graphene-based additives must be produced through reproducible protocols, while assessing their quality through sequences of methods such as those described in the recent ISO/TS 21356-1:2021 standard.^40^ Meanwhile, scalable graphene production approaches, *e.g.*, liquid-phase exfoliation (LPE) methods,^43^ can be adopted to produce graphene dispersions that are easily processed in the form of films and composites by high-throughput and inexpensive printing and compounding techniques compatible with industrial manufacturing chains.^40,44^

In this work, we present a novel approach for improving the electron extraction at the fullerenes/perovskite interface using few-layer graphene flakes (GFs) as PCBM additives. Graphene flakes were industrially produced through wet-jet milling (WJM) exfoliation of graphite,^45^ an LPE method that ensures the massive production of few-layer GFs compliant with ISO/TS 80004 13:2017 and ISO/TS 21356-1:2021 standards.^46^ Once mixed with PCBM to form an ultrathin composite ETL (GF:PCBM) with a few-nanometer thickness (∼10 nm), our flat GFs preferentially orient themselves parallel to the substrate and are uniformly distributed within the polymeric matrix. By combining photoluminescence quantum yield (PLQY), transient photoluminescence (TRPL) and Kelvin probe microscopy (KPFM) measurements, we evince that our GFs locally modify the PCBM energetics for an efficient electron extraction while acting as preferential electron-transporting pathways toward the current collector. Indeed, our triple-cation-based PSCs with GF:PCBM ETLs showed performances superior to those of our reference GF-free device. In particular, the open-circuit voltage (*V*_OC_) and the fill factor (FF) average values improved by +30 meV and +3% points, respectively, upon the incorporation of GFs into PCBM. Our champion GF:PCBM-based PSC achieved a PCE of 20.8%, while showing superior performance stability under continuous illumination conditions compared to reference devices.

## Experimental results

**Scheme 1:**
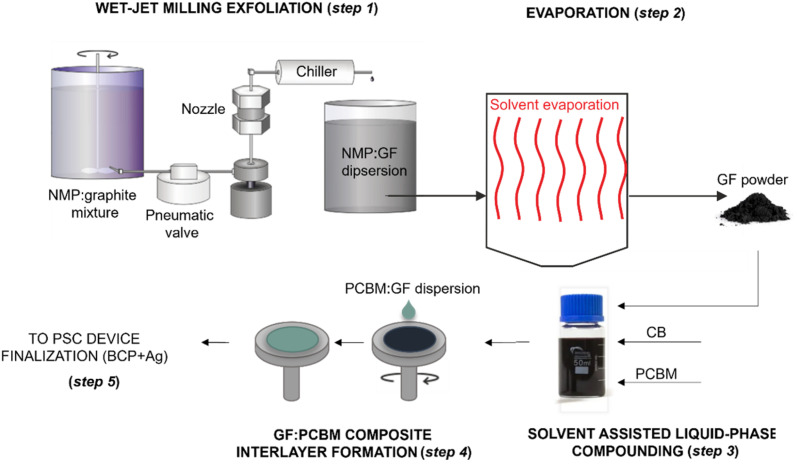
Industrial manufacturing chain for the production of GFs and their exploitation to form the GF:PCBM ETL in the PSC device.

**Fig. 1:**
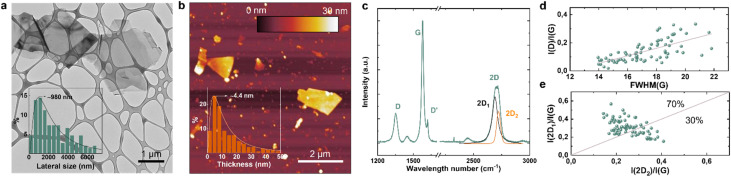
Characterization of WJM-produced GFs. (a) TEM image of representative GFs. The inset panel shows the statistical analysis of the lateral size data (*n* = 200, log-normal fit). (b) AFM image of GFs. The inset panel shows the statistical analysis of the thickness data (*n* = 200, log-normal fit). (c) Representative Raman spectrum of the WJM-produced sample, showing the 2D_1_ and 2D_2_ mode contributions to the 2D mode, resulting from the multi-peak Lorentzian fitting of the 2D mode. (d) *I*(D)/*I*(G) *vs.* FWHM(G) and (e) *I*(2D_1_)/*I*(G) *vs. I*(2D_2_)/*I*(G) plots of the WJM-produced sample. In panel (e), the red line *I*(2D_1_)/*I*(G) = *I*(2D_2_)/*I*(G) represents the few-layer limit condition (∼5 layers).

Scheme 1 shows the manufacturing chain of the GF:PCBM-based PSCs. Experimentally, GFs were produced through a 5-pass WJM protocol (step 1), in which a mixture of graphite and *N*-methyl-2-pyrrolidone (*N*MP) is pressurized at 200 MPa into two jet streams, which then collide into a 0.87 mm-diameter nozzle (WJM apparatus core) to generate shear forces causing the physical exfoliation of the graphite.^45^ As described in previous studies,^41,45,47^ this method enables the massive production (>0.5 kg per day on a single WJM apparatus) of high-quality GFs with an exfoliation yield (defined as the ratio between the weight of the final exfoliated material and the weight of the starting graphite flakes) approaching 100%. Subsequently, the WJM-produced GF dispersion was dried using a BeDimensional's customized drier to remove the NMP (step 2). The GF:PCBM composite ETL was produced by dispersing GFs in chlorobenzene (CB) through ultrasonication (which may also exfoliate graphite residuals), followed by incorporation into PCBM (step 3). Different loadings of GF dispersion were evaluated to obtain 0.5 wt%, 1 wt%, 2.5 wt% and 5 wt% GFs with respect to the PCBM weight (samples hereafter named GF 0.5%, GF 1%, GF 2.5%, and GF 5%). The so-produced GF:PCBM composite dispersions were then deposited onto the triple-cation perovskite to form the ETL (step 4), over which the current collector was finally deposited through vacuum evaporation to complete the PSC (step 5, see also Experimental methods – ESI†). Fig. 1a shows the transmission electron microscopy (TEM) image of representative GFs, featuring irregular flat shapes with straight borders. The lateral size data follow a log-normal distribution peaked at ∼980 nm (Fig. 1a inset). The AFM measurements of the exfoliated samples imaged GFs with thicknesses following log-normal distributions peaked at ∼4.4 nm (Fig. 1b). The Raman spectrum of the WJM-produced GFs (Fig. 1c) shows the typical bands associated with the vibration modes of graphene,^48^*e.g.*, G (∼1580 cm^−1^, associated with the *E*_2g_ phonon mode in the Brillouin zone centre),^49^ D (∼1353 cm^−1^, breathing modes of *K*-point phonons with *A*_1g_ symmetry),^50^ D′ (∼1622 cm^−1^, originating from the intravalley one-phonon double resonance Raman processes, involving one longitudinal optical phonon near the *Γ* point and one defect)^49^ and 2D (∼2710 cm^−1^, attributed to the second order of the D peak).^51,52^Fig. 1d shows the *I*(D)/*I*(G) *vs.* FWHM(G) distribution (*I*(X) indicates the intensity of the X peak; FWHM(G) is the full width at half maximum of the G peak), which is helpful to identify the nature of graphene defects (*e.g.*, edge or structural defects in the basal planes).^51,53^ This plot does not show a linear correlation (*R*^2^ < 0.6), which means that WJM-produced GFs do not exhibit structural defects in their basal planes.^54^ In fact, *I*(D)/*I*(G) positively correlates with the amount of disorder,^55^ while *I*(D)/*I*(G) varies inversely with the crystal size in the absence of defective basal planes.^54^ The thickness of the GFs can be estimated by the analysis of the 2D peak, a complementary approach to AFM analysis. In fact, graphite and few-/multi-layer graphene exhibit a 2D peak having two contributions named 2D_1_ and 2D_2_.^56^ In graphite and multi (>5)-layer graphene, the intensity of the 2D_2_ peak is around twice that of 2D_1_,^57^ while in few-layer graphene, the 2D_1_ peak is more intense than the 2D_2_ peak.^58,59^ As shown in Fig. 1e, the data that fall above the line *I*(2D_1_)/*I*(G) = *I*(2D_2_)/*I*(G) in the *I*(2D_1_)/*I*(G) *vs. I*(2D_2_)/*I*(G) plot are attributed to few-layer GFs, while those below that line correspond to flakes with more than 5 layers. Overall, the morphological and structural characterization indicates that the sample is mainly composed of few-layer graphene (AFM and Raman data) with a lateral size of ∼ 1 μm (TEM data).

**Fig. 2:**
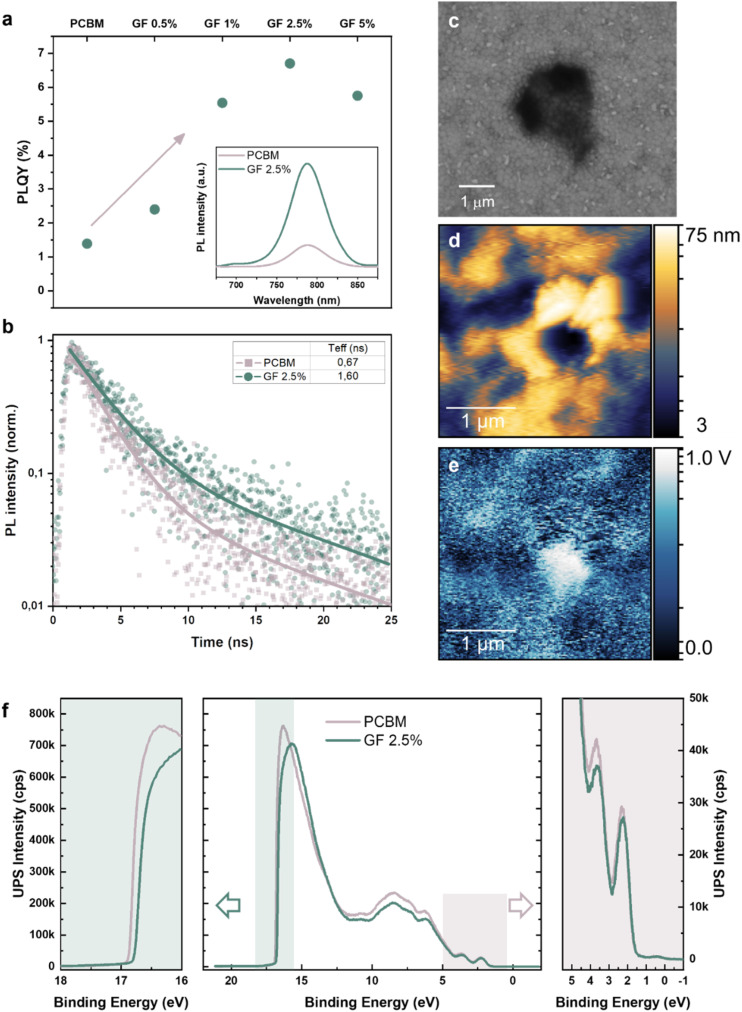
Characterization of the GF:PCBM composite ETLs. (a) PLQY values of triple-cation perovskite/ETL structures (indicated with the name of the ETL), in which the ETL was pristine PCBM or one of the GF:PCBM composites produced with different GFs contents. The inset panel shows the PL spectra of the reference (PCBM) and the most emissive structure (GF 2.5%). (b) TRPL decay of pristine PCBM and GF 2.5% samples at 780 nm using an incident fluence of 100 mW cm^−2^. The samples were irradiated with a pulsed laser centred at 470 nm. (c) Top-view SEM image of the GF 2.5% sample, in which a GF can be distinguished with a lateral dimension of *ca.* ∼1 μm. (d and e) AFM and KPFM images acquired on the same spot of the GF 2.5% sample. At the centre of the images, a GF can be distinguished by its different electronic properties compared to the GF-free background region. (f) Central panel: UPS spectra acquired for pristine PCBM and the GF 2.5% sample; left panel: enlargement of the secondary cut-off region of the UPS spectra; right panel: enlargement of the region near the Fermi level (valence band region) of the UPS spectra.

The so obtained perovskite/ETL structures (referred in the following with the name of the ETL) were first evaluated through PLQY and TRPL measurements. The PLQY analysis (Fig. 2a) revealed a significant enhancement of the emission upon increasing the content of GFs within the PCBM layer, up to a maximum PLQY of 6.8% for the GF 2.5% sample (PLQY = 1.4% for pristine PCBM). The inset of Fig. 2a shows the photoluminescence (PL) spectra of pristine PCBM and of the most emissive structure (GF 2.5% sample). The incorporation of GFs into the PCBM does not change the PL peak position (with a typical emission of the triple-cation perovskite peaked at ∼780 nm), but the PL intensity increases, in accordance with the PLQY trend. Fig. 2b shows the normalized TRPL decays measured at 780 nm for the most emissive structure (GF 2.5%) and for the reference (PCBM), using a fluence of 100 mW cm^−2^. From a stretched exponential analysis, the GF 2.5% sample showed the longest average lifetime. In particular, the average lifetime increased from 0.67 ns in pristine PCBM up to 1.60 ns in GF 2.5%. The PLQY and the TRPL results indicate that the presence of GFs in the PCBM decreases the non-radiative recombination, enhancing both the PLQY and the carrier lifetime. This means that the presence of GFs in the PCBM improves the quality of the perovskite/ETL interface by reducing defect-mediated phenomena and promoting the radiative recombination of carriers, which can be more favourably extracted in a full device. To further elucidate the composite GF:PCBM ETL morphology, the most emissive sample, *i.e.*, GF 2.5%, was studied by atomic force microscopy (AFM). Fig. S1† shows the AFM image obtained for the GF 2.5% sample deposited on a glass substrate, depicting a continuous and homogeneous film with a roughness in the order of 7 nm, in which GFs are distinguishable from the rest of the PCBM matrix. Clearly, the GFs incorporated in the PCBM matrix mainly orient parallelly to the substrate. Since our GFs feature a flat and few-layer morphology, this means that the presence of GFs in PCBM does not substantially alter the dimensional parameters (*e.g.*, thickness) and roughness of the pristine PCBM ETL, avoiding cracks and inhomogeneous thickness. Fig. 2c reports a top-view scanning electron microscopy (SEM) image of the GF 2.5% sample deposited onto a triple-cation perovskite film, focusing on a GF with a lateral dimension on the order of 1 μm. The grainy structure of the perovskite layer is still visible below the GF, confirming the tens of nanometres thickness of the composite ETL even in the GF-rich region. The effect of GFs on the electronic properties of the ETL was evaluated through KPFM measurements performed in a region of the GF 2.5% sample, where a GF was clearly distinguished from the rest of the PCBM matrix (see the AFM image in Fig. 2d). Fig. 2e reports the KPFM image of this region, indicating that the presence of the GF (bright spot) locally modifies the surface potential (*i.e.*, the work function -WF-) of the PCBM matrix (dark areas). Nearby the GF, the work function of the film decreases by more than 0.5 eV. Considering the Fermi level alignment at the materials interface and the metallic behaviour of graphene, our KPFM analysis indicates that the GF-rich regions of PCBM can efficiently collect electrons, which are then transported over the conductive basal planes of GFs towards the current collector. Additional photoelectron spectroscopy (UPS) measurements were performed to further evaluate the effect of GFs on the properties of the ETL. Fig. 2f depicts the UPS spectra obtained for the pristine PCBM and GF 2.5% samples on a spot of ∼55 μm in diameter. The two spectra present a relative energy shift between the two curves of about 0.1 eV. By analysing the secondary electron cut-off region of the UPS spectra (left panel), a WF of 4.3 eV and 4.4 eV was calculated for pristine PCBM and GF 2.5% ETLs, respectively. In the valence band region of the spectra (right panel), the two ETLs do not show any differences, indicating that their highest occupied molecular orbital (HOMO) levels are equally distant from their Fermi level. Overall, these results indicate that the GFs incorporation in PCBM modulates the ETL WF, without shifting the HOMO level with any type of doping.

**Fig. 3:**
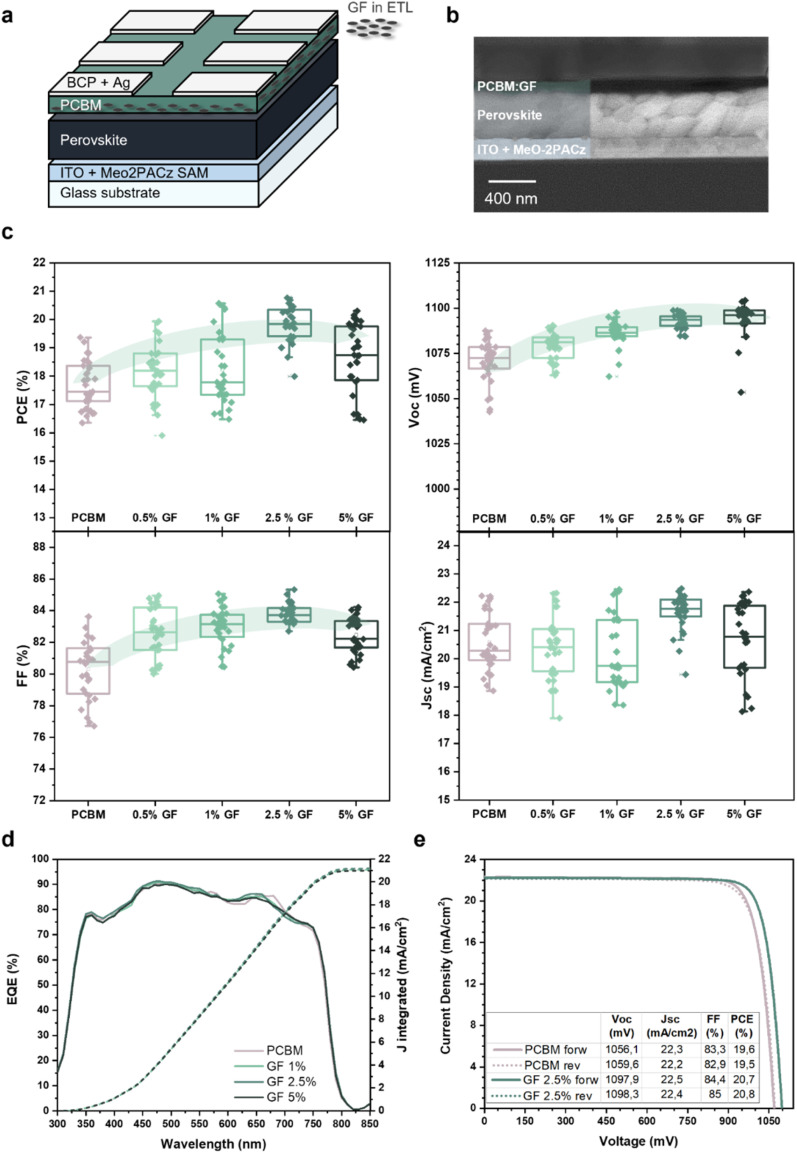
Characterization of the investigated p–i–n PSCs. (a) sketch of the device architecture, based on the GF:PCBM ETL. (b) Cross-sectional SEM image of a GF:PCBM (GF 2.5%) based PSC. (c) Statistical analysis of the photovoltaic parameters measured for 30 different PSCs for each type of configuration: PCE, *V*_OC_, FF and *J*_SC_. (d) EQE and calculated integrated current for the investigated PSC using pristine PCBM and GF:PCBM ETLs. (e) *J*–*V* curve obtained for the pristine PCBM-based and GF 2.5%-based PSCs (reference and champion devices, respectively).

Based on our understanding of the effects of GFs on the properties of PCBM-based ETLs, p–i–n PSCs were fabricated using either the pristine PCBM ETL or the formulated GF:PCBM composite ETLs. Fig. 3a schematically depicts the stack of the investigated p–i–n PSCs, whose fabrication details are reported in the ESI (Experimental methods).† The indium tin oxide (ITO)-coated glass substrate was functionalized with a MeO-2PACz ((2-(3,6-dimethoxy-9*H*-carbazol-9-yl)ethyl)phosphonic acid) self-assembled monolayer, on top of which a triple-cation perovskite with the Cs_0.05_FA_0.79_MA_0.16_Pb(I_0.90_Br_0.10_)_3_ composition (1.60 eV bandgap) was deposited. The top side of the cell was composed of the GF:PCBM (or pristine PCBM) ETLs, topped by bathocuproine (BCP) and evaporated Ag as a current collector. Fig. 3b shows the cross-sectional SEM image of a representative device based on the GF 2.5% ETL, showing a well-defined layered structure. In particular, the image reveals the high crystalline quality of the perovskite layer made of vertical columnar grains, whose morphology is crucial to obtain high-performance PSCs, on top of which a thin (∼10 nm) composite ETL levels the roughness of the underlying perovskite layer. Fig. 3c shows the figures of merit of the reference (pristine PCBM-based) and GF:PCBM-based devices. The device *V*_OC_ and FF undergo an increase upon the incorporation of GFs into PCBM. The *V*_OC_ shows a continuous improvement with increasing GF content, passing from an average value of 1070 mV for the pristine PCBM sample up to 1100 mV for the GF 5% sample, corresponding to a net increase of about 30 mV. The highest FF is instead reached by the GF 2.5% sample, with an impressive maximum value of 85%. By increasing the contents of GFs in PCBM beyond 2.5 wt%, the average FF decreases, likely due to the appearance of inhomogeneities in the GFs distribution along the whole surface. Overall, the trends of *V*_OC_ and FF parameters increment the PCE performances of the tested devices, contributing to a general PCE enhancement compared to the pristine PCBM-based reference cells. The sample with the GF 2.5% ETL reached an average PCE about 2% higher than that of the pristine PCBM-based reference, reaching an average PCE greater than 20%. Concerning the short circuit current (*J*_SC_), the statistical data distribution shows a narrower distribution for the case of GF 2.5%, even though no significant increase of the maximum values was observed. Fig. 3d illustrates the external quantum efficiency (EQE) spectra recorded for the investigated PSCs, excluding any enhancement of the photogenerated current upon the incorporation of GFs in PCBM. Fig. 3e illustrates the *J*–*V* curves and the related photovoltaic parameters for the best-performing pristine PCBM-based reference and GF 2.5%-based devices. According to the previous photovoltaic parameter analyses, while the *J*_SC_ is substantially unvaried, the incorporation of GFs into PCBM causes a significant increase of both *V*_OC_ and FF and a reduced hysteresis of the resulting devices compared to the reference one. Overall, the best GF 2.5%-based PSCs reached a PCE value of 20.8%.

**Fig. 4:**
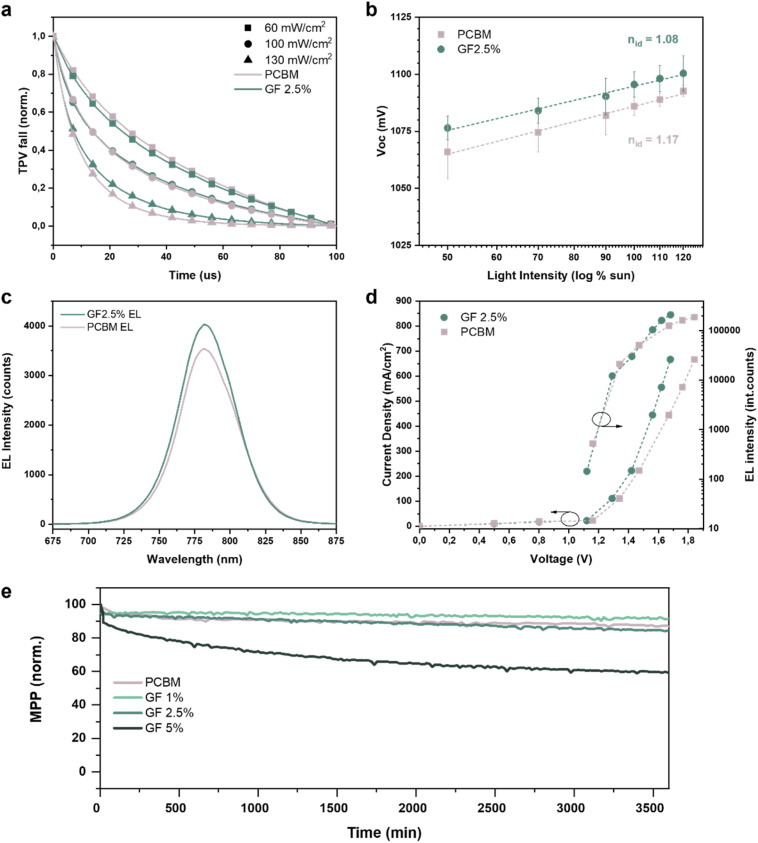
Advanced characterization of the investigated p–i–n PSCs. (a) TPV decay measured for the pristine PCBM- and GF 2.5%-based devices at different incident power densities. (b) *V*_OC_*vs.* light intensity plot obtained for the pristine PCBM- and GF 2.5%-based devices. The dotted line represents the linear fit of the data from which the ideality factor (*n*_id_) can be extrapolated. (c) EL spectra recorded for the pristine PCBM- and GF 2.5%-based devices, while injecting a current density of 660 mA cm^−2^. (d) Current density *vs.* voltage plot obtained for the pristine PCBM- and GF 2.5%-based devices, while being stressed in reverse bias and used as light-emitting diodes (left *y*-axis). Luminance of the light emitted by the devices under different (*J*, *V*) stressing conditions, calculated as the integrals of the recorded EL spectra (right *y*-axis). (e) Maximum power point stability test under continuous illumination of the pristine PCBM-based reference and the GF:PCBM-based PSCs.

Transient photo-voltage (TPV) and transient photo-current (TPC) measurements were performed to reveal the mechanism regulating the device performance enhancement resulting from the incorporation of GFs in the ETL. The measurements were performed on the pristine PCBM-based reference and GF 2.5%-based devices, using different irradiating power densities to simulate different density regimes of charge carriers in the devices. As shown in Fig. 4a, in the low-carrier density regime (60 mW cm^−2^), regulated mainly by radiative recombination processes, the two devices exhibited similar TPV profiles, with the pristine PCBM-based one showing a slightly longer voltage lifetime. Differently, in the high carrier density regime (130 mW cm^−2^), the GF 2.5%-based cell exhibited the longest voltage decay, indicating a longer lifetime of the carriers compared to that of the pristine PCBM-based reference cell. Since in this regime the carrier recombination mechanism is mainly associated with defects and non-radiative processes, it is possible to assert that the presence of GFs at the perovskite/ETL interface reduces the number of recombination events by means of a reduction of the surface recombination velocity of carriers,^60^ further corroborating our *V*_OC_ and PLQY results. Instead, the TPC curves (Fig. S2†) show no significant differences between the two devices indicating similar dynamics of the photogenerated carriers, as expected from the EQE results (Fig. 3d). The role of the GF:PCBM ETL in the device behaviour was further evaluated through *V*_OC_ measurement as a function of light intensity. Fig. 4b illustrates the *V*_OC_*vs.* light intensity plots obtained for the pristine PCBM- and the GF 2.5%-based devices for incident light intensities ranging from 0.5 to 1.2 Sun. The *V*_OC_ data show similar trends for the two devices. Nevertheless, the GF 2.5%-based device shows a lower ideality factor (*n*_id_) compared to that calculated for the pristine PCBM-based reference, further confirming the beneficial effect of the GFs. Thanks to their intrinsic electrical conductivity (resistance measurements of PCMB and GF 2.5% thin films are reported in Fig. S4 and S5 in the ESI†), our data indicate that GFs enhance the carrier extraction rate at the perovskite/ETL interface by creating preferential electron-transporting pathways directed towards the current collector. To validate this hypothesis, we performed additional electroluminescence (EL) measurements on our PSCs. Fig. 4c shows the EL spectra of the pristine PCBM- and GF 2.5%-based devices, while Fig. 4d shows the EL performance as a function of the applied voltage. As expected, the GF 2.5%-based cell shows a stronger emission and more efficient charge injection than the pristine PCBM-based reference. These results confirm our previous findings, proving that the GFs facilitate the electron transport through the perovskite/ETL interface. These findings verify the following dual role of GFs: (i) reducing the density of defects at the ETL/perovskite interface, limiting non-radiative recombination processes; (ii) improving electron injection from the ETL towards the perovskite thanks to the preferential conductive pathways provided by the GFs basal planes (that is translated into more efficient electron extraction in photovoltaic devices).

The effect of GFs embedded into PCBM on the PSC stability was also investigated. Fig. 4e shows the maximum power point (MPP) tracking under continuous illumination in a N_2_ atmosphere for the pristine PCBM- and GF:PCBM-based cells, over a time span of 3800 min. At first, the data revealed a worse stability for the GF 5%-based sample, which also displayed the lowest FF values, indicating the need to optimize the GFs content below a certain threshold. Indeed, lower GFs contents exhibited better stability performances: the GF 2.5%-based sample retained 84.0% of the initial PCE value, comparable with the 86.7% PCE retention of the reference one. The best stability was achieved by the GF 1%-based device, which retained 91.5% of the initial PCE. These results highlight the great accomplishment achieved by the GF:PCBM-based PSCs, showing both PCE and stability enhancement compared to GF-free devices.

## Conclusions

In this work, we demonstrated the potential of embedding graphene into fullerene-based ETLs of inverted p–i–n PSCs. Graphene flakes have been produced with an industrial approach, namely WJM of graphite, leading to exfoliated samples mainly consisting of few-layer GFs with a flat morphology. The latter enable GFs to unalter the dimensional parameters of pristine PCBM upon their incorporation since they tend to orient parallelly to the substrate. Thanks to their electronic properties, the WJM-produced GFs improve the charge extraction properties of PCBM, decreasing the charge recombination rate at the perovskite/ETL interface. As demonstrated by KPFM and UPS analysis, GFs locally modify the PCBM energetics, creating preferential conductive pathways for the electron extraction over their basal planes, resulting in a reduction of non-radiative losses at the perovskite/ETL interface. These effects enhance the performances of the p–i–n triple cation-perovskite-based PSC using PCBM-based ETLs. In particular, compared to pristine PCBM, our GF:PCBM composite ETL improves the *V*_OC_ and FF of the cells, reaching PCE values as high as 20.8% and consistent stability under continuous illumination. These results point out the importance of properly tailoring the interfaces in PSCs, confirming the potential of graphene and related materials as functional additives to boost the charge extraction and device PCE.

## Author contributions

A. Z. and I. B. fabricated the composite matrix and the photovoltaic devices; they performed and assisted in all the characterization studies and analysed the data. F. F. and G. P. performed the optical measurements, M. M. performed the AFM and KPFM analyses. A. Z. visualized and wrote the original draft. S. B. wrote part of the manuscript related to the GFs production, processing, and characterization. M. D. B. and G. G. supervised the activities, analysed the results, and revised the text. A. E. D. C. produced GFs through the WJM method. L. G., S. B., M. I. Z. and G. B. formulated the 15 wt% GF paste. L. G., M. I. Z., J. K. P., G. B., A. E. D. C. and S. B. characterized GFs and analysed the related data. M. P. performed the XPS analysis. S. L. carried out the SEM characterization. G. G. and F. B. acquired the fundings. All the authors reviewed the manuscript.

## Conflicts of interest

There are no conflicts to declare.

## Supplementary Material

TA-011-D2TA07512A-s001
