# Supplementary material for: Enhancing charge extraction in inverted perovskite solar cells contacts via ultrathin graphene:fullerene composite interlayers
Source: J Mater Chem A Mater. 2022 Nov 30;11(24):12866–75. doi: 10.1039/d2ta07512a (PMC10281336; doi:10.1039/d2ta07512a)
Supplement: TA-011-D2TA07512A-s001 [file TA-011-D2TA07512A-s001.pdf]

Electronic Supplementary Material (ESI) for Journal of Materials Chemistry A.
This journal is © The Royal Society of Chemistry 2022

Enhancing Charges Extraction in Inverted Perovskite Solar Cells Contacts via Ultrathin Graphene:Fullerene Composite Interlayers

*Andrea Zanetta[a,±], Isabella Bulfaro[a,±], Fabiola Faini[a], Matteo Manzi[a], Giovanni Pica[a], Michele De Bastiani[a], Sebastiano Bellani[b], Marilena Isabella Zappia[b], Gabriele Bianca[c,d], Luca Gabatel[b,e], Jaya-Kumar Panda[b], Antonio Esaú Del Rio Castillo[b], Mirko Prato[f], Simone Lauciello[g], Francesco Bonaccorso[b], Giulia Grancini[a]**

[a] Department of Chemistry & INSTM, University of Pavia, via T. Taramelli 14, 27100 Pavia, Italy
[b] BeDimensional S.p.A, via Lungotorrente Secca 30R, 16163 Genova, Italy
[c] Graphene Labs, Istituto Italiano di Tecnologia, via Morego 30, 16163 Genova, Italy
[d] Dipartimento di Chimica e Chimica Industriale, Università degli Studi di Genova, via Dodecaneso 31, 16146 Genoa, Italy
[e] Department of Mechanical Engineering – DIME, University of Genova, via Opera Pia 15, 16145 Genova, Italy
[f] Materials Characterization Facility, Istituto Italiano di Tecnologia, via Morego 30, 16163 Genova, Italy
[g] Electron Microscopy Facility, Istituto Italiano di Tecnologia, via Morego 30, 16163 Genova, Italy
[±] Equally contributing authors
*Corresponding author: giulia.grancini@unipv.it

Supporting Information

Experimental Methods

Solvents were purchased from Sigma-Aldrich (highest purity and anhydrous); PbI_2, $PbBr_2$, CsI (99.99% purity) and MeO-2PACz ([2-(3,6-Dimethoxy-9H-carbazol-9-yl)ethyl]phosphonic Acid) were purchased from TCI; organic halides salts were purchased from GreatCell Solar. (6,6)-Phenyl C61 butyric acid methyl ester (PCBM, >99.5%) were purchased from Lumtec; Bathocuproine (BCP, sublimed grade, 99.99% purity). The dispersion of graphene flakes (GFs) (15 wt%) in chlorobenzene was supplied by BeDimensional S.p.A. (for the purpose of this work, the production of this product is also reported in this document). Unless otherwise specified, materials and solvents have been used as received. All the solutions/dispersions were prepared into an Ar-filled glovebox and the spin coating steps were carried out into a N_2-filled glovebox (in both case, O_2 concentration <0.1 ppm and H_2O concentration < 0.1 ppm)

Graphene flakes (powder and dispersion) production

The GFs were produced through wet-jet milling (WJM) method, using a 5-pass protocol. [1,2] Experimentally, a mixture of graphite (+100 mesh, Sigma Aldrich) and N-Methyl-2-pyrrolidone (NMP) (> 97%, Sigma Aldrich) was prepared in a container and mixed by a mechanical stirrer (Eurostar digital Ika-Werke). The mixture was then pressurized at 200 MPa into two jet streams, which then collide into a 0.87 mm-diameter nozzle (WJM *apparatus* core). The turbulence of the solvent generates the shear forces causing the physical exfoliation of the graphite.[2] The exfoliated sample was cooled by means of a chiller and then reprocessed 5 time on sequential WJM *apparati*. The as-produced WJM-produced GF dispersion was dried in a BeDimensional's customized drier to remove the NMP. The dried GF powder was redispersed in anhydrous chlorobenzene (CB) (99.8%, Sigma Aldrich) with a concentration of 15 wt%.

Graphene characterization

Bright-field transmission electron microscopy (BF-TEM) images were acquired with a JEM 1011 (JEOL) TEM (thermionic W filament), operating at 100 kV. The samples were prepared by drop-casting the WJM-produced GF dispersion onto ultrathin C-coated Cu grids. The samples were rinsed with deionized water and subsequently dried overnight under vacuum before measurements. Atomic force microscopy (AFM) images were acquired with a NX10 AFM (Park System, Korea) by means of a non-contact cantilever PPP-NCHR 10 M (Nanosensors, Switzerland) having a tip diameter inferior to 10 nm, a resonance frequency of ~330 kHz, and a force constant of 42 N m^{-1}. The images were collected using a non-contact mode, keeping the working setpoint above 70% of the free oscillation amplitude. The scan rate for the acquisition of the images was 0.2 Hz. The samples were prepared by drop-casting a 1:10 diluted WJM-produced GF dispersion onto mica sheets (G250-1, Agar Scientific Ltd.), followed by heating at 100 °C for 15 min to dry the sample. Raman spectroscopy measurements were carried out by means of a Renishaw microRaman inVia 1000 mounting an objective with 0.9 numerical aperture (NA), using an excitation wavelength of 514 nm and an incident power of 1 mW. The samples were prepared by drop-casting the WJM-produced GF

dispersion onto Si/SiO_2 substrates and subsequently dried under vacuum. 100 spectra were collected to perform the statistical analysis.

Perovskite solar cell fabrication

Prepatterned ITO/glass substrates were sequentially cleaned with acetone and 2-propanol (IPA) by ultrasonication for 15 min in each solvent. The ITO/glass substrates were then dried with N_2 and treated with oxygen plasma at 100 mW for 10 min. The substrates were moved into the glovebox for subsequent layers deposition. The MeO-2PACz self-assembly monolayers were deposited by spin coating (3000 rpm, 30 s, 1500 rpm/s) from a 0.335 mg/mL solution in ethanol, and then annealed at 100 °C for 10 min. The perovskite precursor solution (1.1 M) was prepared with the mixed cations (Pb, Cs, FA, and MA) and halides (I and Br) composition, dissolved in a solvent mixture (DMF/DMSO = 4/1 vol:vol) according to the formula Cs $Cs_{0.05}FA_{0.79}MA_{0.16}Pb(I_{0.90}Br_{0.10})_3$ with an excess of PbI_2 of 1 at%. The perovskite layer was deposited *via* a two-step spin-coating procedure with 1000 rpm for 12 s and 5000 rpm for 27 s. Chlorobenzene antisolvent was dripped on the spinning substrate during the 21 s of the second spin-coating step. Subsequently, the samples were annealed at 100 °C for 30 min. On top of the perovskite, either the pristine PCBM or GF:PCBM ETL layer was deposited. The PCBM solution was prepared with a 20 mg/mL concentration in chlorobenzene; in case of the GF:PCBM composite dispersions, different concentrations of GFs were evaluated to obtain 0.5 wt%, 1wt%, 2.5wt% and 5wt% of GFs relatively to the PCBM weight (samples hereafter named GF 0.5%, GF 1%, GF 2.5%, and GF 5%). These dispersion were prepared by diluting the 15 wt% GF paste supplied by BeDimensional S.p.A. The 1.5 wt% GF dispersion was prepared by diluting the 15 wt% GF paste supplied by BeDimensional S.p.A.; before use, all the GF dispersions were sonicated for 6 h. The ETLs were deposited by dynamic spin coating procedure, with a single step program (2000 rpm, 30 s, 1000 rpm/s), followed by 10 s annealing at 100 °C. Lastly, the thin layers of BCP (0.5 mg/mL in IPA) were spin-coated at 4000 rpm for 30 s, with a ramping rate of

1000 rpm/s. The devices were then completed by depositing 80 nm-thick Ag back electrode through vacuum thermal evaporation.

Photovoltaic device characterization

Current density voltage measurements were performed in ambient conditions under simulated AM 1.5 light with an intensity of 100 mW cm^{-2} (Wavelabs-Sinus 70). The intensity was calibrated using a Si reference cell. Cells were scanned using a Keithley 2450 source-meter backward and forward from 1.2 V to -0.1 V, with a scanning velocity of 100 mV/s. The pixel area was 3 mm by 1.5 mm. The stability measurements were performed under N2 inert atmosphere and ambient temperature using a stability box (Litos Lite) from Fluxim, coupled with a Wavelabs solar simulator continuously illuminating the devices. The light spectrum used was the standard AM1.5G one, monitored during the whole experiment by the spectrophotometer integrated with the solar simulator system. The temperature reached by the devices was monitored by the thermocouples integrated within the stability box system, achieving a maximum temperature about 33°C due to the irradiation heat provided by the light source. The external quantum efficiency (EQE) and transient measurements (transient photocurrent and transient photovoltage) were performed using ARKEO platform (Cicci Research S.r.l.).

Photoluminescence, Transient Photoluminescence and Electroluminescence measurements

The photoluminescence spectra of the different samples were collected using a customized optical setup,[3] in which the excitation was provided by pulsed/CW laser at 470 nm (PicoQuant), while the luminescence was analysed with an interferometer (GEMINI by Nireos) and recorded with a single photon detector (IDQuantique) coupled with a Time Tagger (Swabian Instrument). The electroluminescence (EL) analysis was performed using the ARKEO platform for the acquisition of the emitted light spectrum, coupled with a Keithley 2450 source-meter as the power supply unit to the devices. The devices were stressed by different and discrete current values in order to inject an

equal number of charge carriers for each different device tested; the EL signal was then recorder by the spectrometer integrated in the optical line of the ARKEO.

Atomic Force Microscopy and Scanning Electron Microscopy

Atomic forxe microscopy (AFM) and Kelvin probe force microscopy (KPFM) measurements have been performed by a TriA AFM instrument from A.P.E.Research. Scanning electron microscopy (SEM) analysis was performed by Field Emission Scanning Electron Microscopy (FE-SEM) using a JEOL JSM-7500 FA (Jeol, Tokyo, Japan), operating at 10 kV acceleration voltage and considering back-scattering electrons for enhancing compositional differences.

Ultraviolet photoelectron spectroscopy - UPS

UPS measurements were performed on a Kratos Axis UltraDLD spectrometer using a He I (21.22 eV) discharge lamp, on an area of 55 μm in diameter, at pass energy of 10 eV, energy step of 0.025 eV and dwell time of 100 ms. The work function (*i.e.*, the energy difference between the energy of an electron at rest in the vacuum nearby the surface of the material and the Fermi level of the material) was measured from the threshold energy for the emission of secondary electrons during He I excitation. A −9.0 V bias was applied to the sample to precisely determine the high binding energy cut-off, as discussed in previous work[4]. The position of the highest occupied molecular orbital (HOMO) level of PCBM *vs.* vacuum level was estimated by measuring the position of the low binding energy cut-off with respect to the Fermi level (*i.e.*, the zero of the binding energy scale).[5] The obtained value was then added to the work function one.

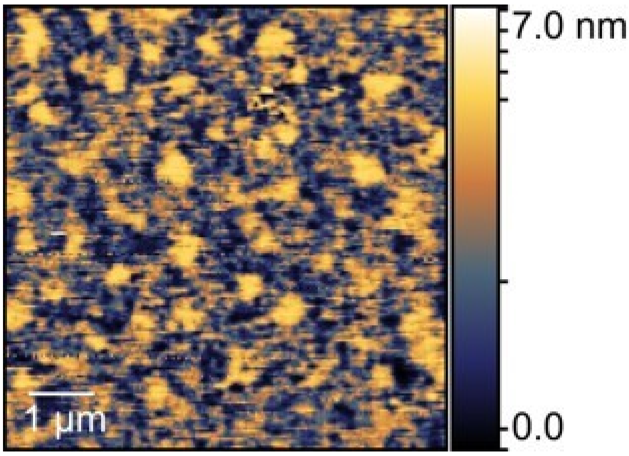

Figure S1. AFM morphology image of a GF 2.5% film deposited on glass. The sample shows a flat morphology, in which GFs can be distinguished within the PCBM matrix. The GFs show lateral dimensions in the order of 500 nm and are oriented parallel to the substrate.

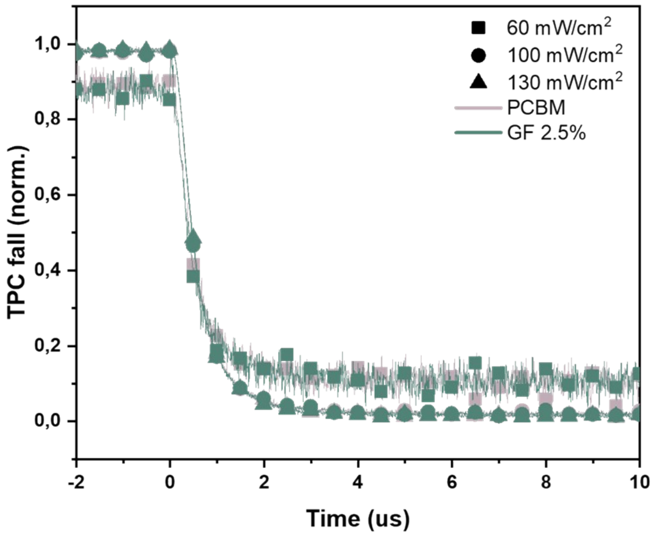

Figure S2. Transient photo-current decay measured for the pristine PCBM- and GF 2.5%-based PSCs for different power densities of the incident irradiation. The TPC decays do not show significant differences (neither at different injected carrier regimes), in accordance with EQE and J_{SC} analysis.

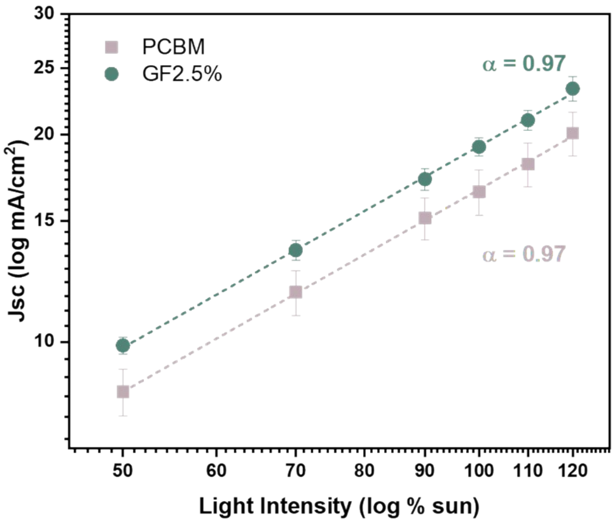

Figure S3. J_{SC} *vs.* light intensity plot measured for the pristine PCBM- and GF 2.5%-based PSCs. Both devices show a linear trend with an α value about 0.97, as expected for highly performant PSCs devices.

Resistance measurements on PCBM and GF 2.5% thin films

Devices based on PCBM and GF 2.5% thin films were fabricated on a Si substate on top of which an interdigitated Au back electrode was deposited (see the sketch of Figure S4). The Si-Au substrates have been washed by sequential ultrasonic bath cleaning in acetone and isopropyl alcohol, followed by 15 min UV-ozone treatment. After that, the PCBM and GF 2.5% thin films have been deposited by a dynamic spin-coating process, following the same procedure used for the photovoltaic devices' ETL. The so-produced devices were tested by applying a voltage scan from -4V to +4V and measuring the current flowing in the device. The scan rate was 250 mV/s. Figure S5 shows the I-V curves obtained for the pristine PCBM- and the GF 2.5%-based devices, revealing that the GF 2.5%-based device shows a current higher than that of the PCBM pristine counterpart. These data confirm that the incorporation of GFs into PCBM increases the electrical conductivity of the pristine polymer. In particular, the linear trend of the current in the films with the applied voltage can be fitted with the Ohm's laws to estimate the device resistance (R). Based on this analysis, the GF 2.5%-based device shows a reduced R of 1.87 GΩ compared to the pristine PCBM-based one, whose R value is 3.83 GΩ.

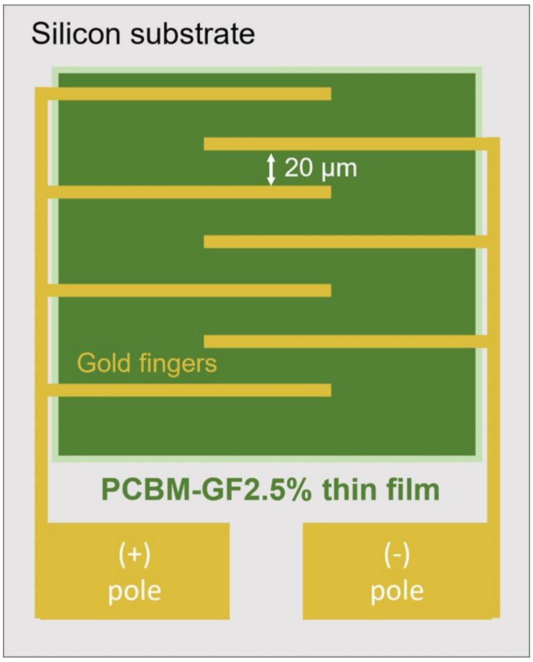

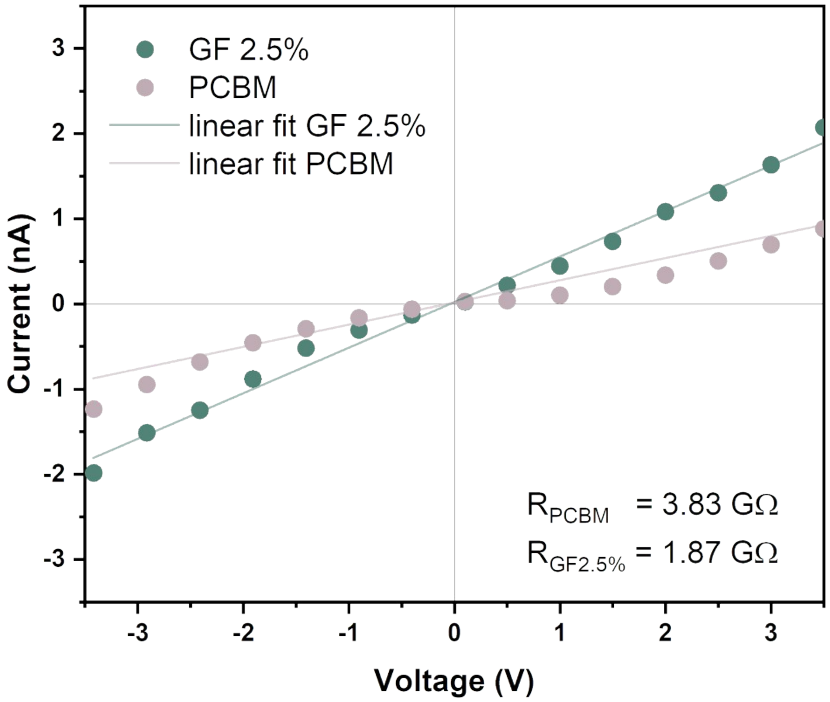

Figure S4. Schematic representation of the devices fabricated for the R measurements.

Figure S5. I-V characteristic curves measured for the pristine PCBM and GF2.5% devices. The linear fitting of the curves allows the computation of the value of R for the two different materials, as reported in the graph

References:

1 S. Bellani, E. Petroni, A. E. Del Rio Castillo, N. Curreli, B. Martín-García, R. Oropesa-Nuñez, M. Prato and F. Bonaccorso, *Advanced Functional Materials*, 2019, **29**, 1807659.

2 A. E. D. R. Castillo, V. Pellegrini, A. Ansaldo, F. Ricciardella, H. Sun, L. Marasco, J. Buha, Z. Dang, L. Gagliani, E. Lago, N. Curreli, S. Gentiluomo, F. Palazon, M. Prato, R. Oropesa-Nuñez, P. S. Toth, E. Mantero, M. Crugliano, A. Gamucci, A. Tomadin, M. Polini and F. Bonaccorso, *Mater. Horiz.*, 2018, **5**, 890–904.

3 G. Pica, D. Bajoni and G. Grancini, *Structural Dynamics*, 2022, **9**, 011101.

4 M. G. Helander, M. T. Greiner, Z. B. Wang and Z. H. Lu, *Applied Surface Science*, 2010, **256**, 2602–2605.

5 P. Schulz, E. Edri, S. Kirmayer, G. Hodes, D. Cahen and A. Kahn, *Energy Environ. Sci.*, 2014, **7**, 1377–1381.

6 J.-B. Wu, M.-L. Lin, X. Cong, H.-N. Liu and P.-H. Tan, *Chem. Soc. Rev.*, 2018, **47**, 1822–1873.

7 S. Bellani, L. Najafi, B. Martín-García, A. Ansaldo, A. E. Del Rio Castillo, M. Prato, I. Moreels and F. Bonaccorso, *J. Phys. Chem. C*, 2017, **121**, 21887–21903.

8 S. Bellani, L. Najafi, M. Prato, R. Oropesa-Nuñez, B. Martín-García, L. Gagliani, E. Mantero, L. Marasco, G. Bianca, M. I. Zappia, C. Demirci, S. Olivotto, G. Mariucci, V. Pellegrini, M. Schiavetti and F. Bonaccorso, *Chem. Mater.*, 2021, **33**, 4106–4121.
